# Preventive Administration of Non-Allergenic Bet v 1 Peptides Reduces Allergic Sensitization to Major Birch Pollen Allergen, Bet v 1

**DOI:** 10.3389/fimmu.2021.744544

**Published:** 2021-10-26

**Authors:** Oluwatoyin Akinfenwa, Huey-Jy Huang, Birgit Linhart, Margarete Focke-Tejkl, Susanne Vrtala, Alina Poroshina, Alexandra Nikonova, Musa Khaitov, Nicholas J. Campion, Julia Eckl-Dorna, Verena Niederberger-Leppin, Bernhard Kratzer, Peter Anton Tauber, Winfried F. Pickl, Michael Kundi, Raffaela Campana, Rudolf Valenta

**Affiliations:** ^1^ Division of Immunopathology, Department of Pathophysiology and Allergy Research, Center for Pathophysiology, Infectiology and Immunology, Medical University of Vienna, Vienna, Austria; ^2^ Karl Landsteiner University of Health Sciences, Krems, Austria; ^3^ National Research Center (NRC) – Institute of Immunology Federal Medical-Biological Agency (FMBA) of Russia, Moscow, Russia; ^4^ Immunology Department, Pirogov Russian National Research Medical University, Moscow, Russia; ^5^ Department of Otorhinolaryngology, Medical University of Vienna, Vienna, Austria; ^6^ Institute of Immunology, Center for Pathophysiology, Infectiology & Immunology, Medical University of Vienna, Vienna, Austria; ^7^ Institute for Hygiene and Applied Immunology, Centre for Public Health, Medical University of Vienna, Vienna, Austria; ^8^ Laboratory for Immunopathology, Department of Clinical Immunology and Allergy, Sechenov First Moscow State Medical University, Moscow, Russia

**Keywords:** allergy, Bet v 1, T cell epitope-containing peptides, allergen-specific tolerance induction, allergy prophylaxis, prevention, mouse model

## Abstract

IgE-mediated allergy to birch pollen affects more than 100 million patients world-wide. Bet v 1, a 17 kDa protein is the major allergen in birch pollen responsible for allergic rhinoconjunctivitis and asthma in birch pollen allergic patients. Allergen-specific immunotherapy (AIT) based on therapeutic administration of Bet v 1-containing vaccines is an effective treatment for birch pollen allergy but no allergen-specific forms of prevention are available. We developed a mouse model for IgE sensitization to Bet v 1 based on subcutaneous injection of aluminum-hydroxide adsorbed recombinant Bet v 1 and performed a detailed characterization of the specificities of the IgE, IgG and CD4^+^ T cell responses in sensitized mice using seven synthetic peptides of 31-42 amino acids length which comprised the Bet v 1 sequence and the epitopes recognized by human CD4^+^ T cells. We then demonstrate that preventive systemic administration of a mix of synthetic non-allergenic Bet v 1 peptides to 3-4 week old mice significantly reduced allergic immune responses, including IgE, IgG, IgE-mediated basophil activation, CD4^+^ T cell and IL-4 responses to the complete Bet v 1 allergen but not to the unrelated major grass pollen allergen Phl p 5, without inducing Bet v 1-specific allergic sensitization or adaptive immunity. Our results thus demonstrate that early preventive administration of non-allergenic synthetic T cell epitope-containing allergen peptides could be a safe strategy for the prevention of allergen-specific IgE sensitization.

## Introduction

Immunoglobulin E (IgE)–associated allergy is the most frequent immunologically mediated hypersensitivity disease and affects more than 30% of the world´s population (1). Allergic patients suffer from a variety of clinical symptoms which include hay fever (i.e., rhinitis, conjunctivitis), asthma, skin inflammation, gastrointestinal allergy and life-threatening systemic anaphylactic shock (2). The detailed analysis of the evolution of allergic sensitization from early childhood to adolescence suggests that allergic sensitizations occur early in life and, depending on genetic and environmental factors, in particular in response to repeated allergen contact, progresses from clinically silent forms of IgE sensitization towards symptomatic allergy (3). Symptomatic allergy also often starts with mild symptoms such as allergic rhinitis and then progress to severe forms such as allergic asthma (4). Therefore, allergy may be compared with other major diseases affecting mankind such as cancer, cardiovascular diseases, autoimmune diseases and metabolic diseases which evolve initially as a clinically inapparent form which can be only detected by preventive medical examination in phenotypically still “healthy” subjects. Similar as for the aforementioned non-communicable diseases which heavily affect mankind and create a huge burden to the health care systems, early preventive intervention strategies are needed for allergy (5). Preventive strategies for allergy include besides attempts of general immunomodulation also allergen-specific and non-specific forms of prevention, for example early allergen-specific immunotherapy, AIT, allergen avoidance, allergen-specific tolerance induction and application of immunomodulating substances such as pre- and probiotics (6–15).

The concept that synthetic T cell epitope-containing peptides could be used for tolerance induction in allergy has been introduced by several research groups (16–18). The approach has then been mainly pursued for AIT of already established allergy in clinical trials but not so much for specific primary prevention of allergy (19). There are several challenges which need to be met if one considers the use of allergen-derived peptides for prevention of allergy. First of all, the clinically most relevant allergens must be defined and a mix of non-allergenic peptides (i.e., peptides which do not induce allergic sensitization) and comprise the T cell epitope repertoires of humans must be defined.

In this study, we evaluated the approach of using relatively long synthetic T cell epitope-containing peptides (i.e., peptides longer than 30 amino acids) for preventive tolerance induction. The possible advantage of using such “long” synthetic peptides could be that fewer peptides are needed to include the relevant T cell epitopes of the allergen, but care must be taken that the peptides are not too long to become immunogenic for B cells and to eventually induce an IgE-dependent allergic immune response. The use of long peptides for AIT has been suggested earlier but their usefulness for prevention has not been extensively studied (20–23). We have previously shown that the use of “long” peptides can be used to cover the human T cell epitope repertoire even of complex allergen sources such as house dust mites which contain several important allergens (24). For our proof of principle study, we focused on the major allergen of birch, Bet v 1, a 17 kDa protein belonging to the group of pathogenesis-related PR10 proteins (25). Bet v 1 is the key molecule responsible for birch pollen allergy and allergic cross-sensitization to Bet v 1-related PR10 molecules in pollen of early flowering trees (e.g., alder, hazel, hornbeam, oak) and in a variety of plant-derived foods (i.e., fruits, vegetables and spices) (26). More than 100 million patients, in particular in Northern Europe, Russia but also other parts of the world are sensitized to Bet v 1 and the evolution of IgE sensitization to Bet v 1 and its clinical relevance has been analyzed in birth cohorts and cross-sectional studies, respectively (27, 28). Tolerance induction to Bet v 1 with the complete recombinant allergen, recombinant hypoallergenic Bet v 1 derivatives and with a short immunodominant peptide have been studied in murine models but several important questions have not yet been addressed (29–31). For example, the effect of a low dose mix of “long” Bet v 1 peptides comprising the T cell epitope repertoire of humans has not been evaluated. In particular, it has not been studied if such a peptide mix can induce tolerance at the level of IgE, IgG, CD4^+^ T cells and cytokines without *per se* inducing allergic sensitization or any adaptive immunity. Furthermore, it has not been studied if the administration of Bet v 1 peptides is specific for Bet v 1 or if there could be a bystander effect for an immunologically unrelated allergen.

## Materials and Methods

### Allergens, Synthetic Peptides

Purified recombinant Bet v 1 and Phl p 5 were purchased from Biomay AG (Vienna, Austria). Seven Bet v 1-derived long peptides (L1-L7) with a length of 31 to 42 amino acids spanning the entire sequence of Bet v 1 (**Table 1**) and seventeen Bet v 1-derived short peptides (S1-S17) of 12 amino acids in length spanning amino acids 85 to 160 of Bet v 1 (**Table 1**) were produced by chemical synthesis using Fmoc amino acid protection and HBTU coupling on a peptide synthesizer (Liberty Blue, CEM Corporation, USA). The short peptides were overlapping for at least 5 amino acids. The seven long Bet v 1 peptides (L1-L7) are similar to a set of peptides reported to lack IgE reactivity and allergenic activity in birch pollen allergic patients but were optimized to span the complete Bet v 1 sequence (32). The 14mer peptide BV139 (MGETLLRAVESYLL) which has been previously described as major T cell epitope in Bet v 1-sensitized BALB/c mice (31) is part of the long peptides L6 and L7, whereas peptide L5 contains only the first six amino acids of BV139. Likewise, the short peptides do not contain complete BV139. S14 and S15 contain ten amino acids of BV139 and S13 as well as S16 only 6 amino acids thereof. Peptides were purified by HPLC (Dionex UltiMate 3000; Thermo Fisher Scientific) to at least 90% purity and their molecular weights confirmed by MALDI-TOF mass spectrometry (Microflex, Bruker, Billerica, MA) as described (32). Lyophilised peptides were stored at -20°C and reconstituted in endotoxin-free water for use. The Bet v 1-derived synthetic peptide sequences and their biochemical characteristics are summarized in **Table 1**. The ability of the six long Bet v 1 peptides P1-P6 to stimulate CD4^+^ T cell responses in blood samples obtained before, during and after the birch pollen season in a population of highly birch pollen-exposed subjects (birch pollen allergic patients: n=6; patients with allergy without birch sensitization: n=4; non-allergic subjects: n=9) from the Moscow region in Russia was studied by carboxyfluorescein diacetate succinimidyl ester (CFSE) dilution assay using two different concentrations (1.2nM, 12nM) as described (24). Together the six peptides P1-P6 comprised the Bet v 1-specific human CD4^+^ T cell epitopes as tested in the Moscow population (**Supplementary Figure 1**).

**Table 1:** Characteristics of Bet v 1-derived synthetic peptides.

Bet v 1-derived long peptides					
**Peptide**	**Position aa**	**Sequence**	**Number of aa**	**MW (DA)**	**pI**
** L1**	1 - 35	MGVFNYETETTSVIPAARLFKAFILDGDNLFPKVA	35	3876.48	4.78
** L2**	30 - 66	LFPKVAPQAISSVENIEGNGGPGTIKKISFPEGFPFK	37	3902.51	8.43
** L3**	61 - 96	EGFPFKYVKDRVDEVDHTNFKYNYSVIEGGPIGDTL	36	4150.57	4.65
** L4**	91 - 126	PIGDTLEKISNEIKIVATPDGGSILKISNKYHTKGD	35	3738.3	8.73
** L5**	110 - 145	DGGSILKISNKYHTKGDHEVKAEQVKASKEMGETLL	36	3942.46	6.93
** L6**	119 - 160	NKYHTKGDHEVKAEQVKASKEMGETLLRAVESYLLAHSDAYN	42	4762.29	6.30
** L7**	130 - 160	KAEQVKASKEMGETLLRAVESYLLAHSDAYN	31	3452.88	5.59
**Bet v 1-derived short peptides**					
**Peptide**	**Position aa**	**Sequence**	**Number of aa**	**MW (DA)**	**pI**
** S1**	85 - 96	VIEGGPIGDTLE	12	1199.32	3.57
** S2**	89 - 100	GPIGDTLEKISN	12	1243.38	4.37
** S3**	93 - 104	DTLEKISNEIKI	12	1402.61	4.68
** S4**	97 - 108	KISNEIKIVATP	12	1312.57	8.59
** S5**	101 - 112	EIKIVATPDGGS	12	1186.33	4.37
** S6**	105 - 116	VATPDGGSILKI	12	1170.37	5.81
** S7**	109 - 120	DGGSILKISNKY	12	1294.47	8.50
** S8**	113 - 124	ILKISNKYHTKG	12	1401.67	10.00
** S9**	117 - 128	SNKYHTKGDHEV	12	1414.50	6.66
** S10**	121 - 132	HTKGDHEVKAEQ	12	1378.46	6.01
** S11**	125 - 136	DHEVKAEQVKAS	12	1340.46	5.45
** S12**	129 - 140	KAEQVKASKEMG	12	1305.51	8.50
** S13**	133 - 144	VKASKEMGETLL	12	1305.55	6.11
** S14**	137 - 148	KEMGETLLRAVE	12	1375.60	4.79
** S15**	141 - 152	ETLLRAVESYLL	12	1406.64	4.53
** S16**	145 - 156	RAVESYLLAHSD	12	1360.49	5.32
** S17**	149 - 160	SYLLAHSDAYN	11	1253.33	5.06
**Previously described Bet v 1-derived peptides (32)**					
**Peptide**	**Position aa**	**Sequence**	**Number of aa**	**MW (DA)**	**pI**
** P1**	1 - 24	MGVFNYETETTSVIPAARLFKAFIC	25	2809.3	6.28
** P2**	30 - 59	LFPKVAPQAISSVENIEGNGGPGTIKKISFC	31	3202.7	8.63
** P3**	50 - 79	CGPGTIKKISFPEGFPFKYVKDRVDEVDHTN	31	3525	7.00
** P4**	110 - 139	DGGSILKISNKYHTKGDHEVKAEQVKASKEC	31	3400.8	8.44
** P5**	130 - 160	CKAEQVKASKEMGETLLRAVESYLLAHSDAYN	32	3556.1	5.49
** P6**	75 - 104	CVDHTNFKYNYSVIEGGPIGDTLEKISNEIK	31	3484.9	4.70
**Alignment of Bet v 1 peptides L1 - L7 and P1-P6**					
** **	**Position aa**	**Sequence**	** **	** **	** **
**Bet v 1**	1 - 80	MGVFNYETETTSVIPAARLFKAFILDGDNLFPKVAPQAISSVENIEGNGGPGTIKKISFPEGFPFKYVKDRVDEVDHTNF			
** L1**	1 - 35	MGVFNYETETTSVIPAARLFKAFILDGDNLFPKVA			
** P1**	1 - 24	MGVFNYETETTSVIPAARLFKAFIC			
** L2**	30 - 66	LFPKVAPQAISSVENIEGNGGPGTIKKISFPEGFPFK			
** P2**	30 - 59	LFPKVAPQAISSVENIEGNGGPGTIKKISFC			
** L3**	61 - 96	EGFPFKYVKDRVDEVDHTNF			
** P3**	50 - 79	CGPGTIKKISFPEGFPFKYVKDRVDEVDHTN			
** P6**	75 - 104	CVDHTNF			
**Bet v 1**	81 - 160	KYNYSVIEGGPIGDTLEKISNEIKIVATPDGGSILKISNKYHTKGDHEVKAEQVKASKEMGETLLRAVESYLLAHSDAYN			
** L3**	61 - 96	KYNYSVIEGGPIGDTL			
** P6**	75 - 104	KYNYSVIEGGPIGDTLEKISNEIK			
** L4**	91 - 126	PIGDTLEKISNEIKIVATPDGGSILKISNKYHTKGD			
** P4**	110 - 139	DGGSILKISNKYHTKGDHEVKAEQVKASKEC			
** L5**	110 - 145	DGGSILKISNKYHTKGDHEVKAEQVKASKEMGETLLRAVESYLL			
** P5**	130 - 160	CKAEQVKASKEMGETLLRAVESYLLAHSDAYN			
** L6**	119 - 160	NKYHTKGDHEVKAEQVKASKEMGETLLRAVESYLLAHSDAYN			
** L7**	130 - 160	KAEQVKASKEMGETLLRAVESYLLAHSDAYN			

### Mice

Female BALB/c mice of different age groups (3-4 weeks for tolerance induction experiments, 6-8 weeks for immunization experiments) were purchased from Charles River (Sulzfeld, Germany) and were maintained according to the guidelines for animal welfare. All experimental procedures were approved by the Animal Ethics Committee of the Medical University of Vienna and the Austrian Federal Ministry of Science, Research and Economy BMWFW (66.009/0431-V/3b/2019). Experiments were performed in accordance with national and international guidelines of laboratory animal care.

### Sensitization of Mice With Bet v 1

Groups of mice, 6 to 8 weeks of age (n = 5), were sensitized by a single subcutaneous (s.c.) injection with 5µg, 10µg, 20µg or 40µg of recombinant Bet v 1 adsorbed to 75μl of aluminum hydroxide (Al(OH)_3_) (Alu-Gel-S, SERVA, Heidelberg, Germany) and made up to a final injection volume of 150μl per mouse with PBS or 75μl of PBS and 75μl of Al(OH)_3_ alone (negative control). Blood samples were taken from the tail veins before sensitization and at days 14, 28 and 42 and serum was prepared by centrifugation. Specific IgE sensitization was determined by the measurement of allergen-specific IgE antibodies (33, 34).

For mapping of epitopes recognized by CD4^+^ T cell epitopes, groups of mice, 6 to 8 weeks of age (n = 6), received three s.c. injections of 10µg or rBet v 1 adsorbed to Al(OH)_3_ every two weeks (days 0, 14 and 28). Blood samples were taken from the tail veins before sensitization as well as on days 20 and 34 to measure Bet v 1-specific antibody responses. Mice were sacrificed 7 days after the last sensitization (day 35) and spleen cells were isolated from each mouse and pooled for cellular analyses (33). More pronounced T cell responses were obtained when mice received 2 sensitizations with Bet v 1 10 weeks apart and were sacrificed at week 25 (12 weeks after last sensitization).

The mapping of CD4^+^ T cell epitopes was performed in three independent experiments obtaining identical results.

For mapping of epitopes recognized by IgE and IgG antibodies, groups of mice, 6 to 8 weeks of age (n =10), received three s.c. injections of 10µg or rBet v 1 adsorbed to Al(OH)_3_ every two weeks (days 0, 14 and 28). Blood samples were taken from the tail veins before sensitization as well as on days 20, 34, 42 and 56 for ELISA analysis. The mapping of antibody epitopes was performed in two independent experiments and gave identical results.

### Administration of Bet v 1 Peptides for Preventive Induction of Specific Tolerance in Mice

Three- to four-week-old female Balb/c mice were used in the tolerance induction experiments.

**Figure 1:**
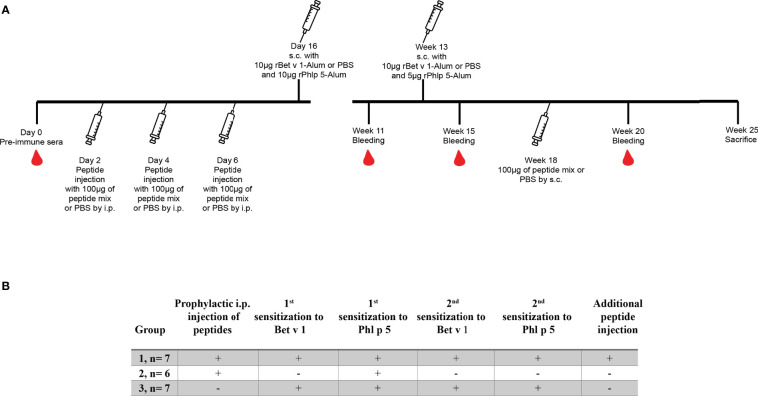
Scheme for first preventive treatment with Bet v 1-derived peptides. **(A)** Three to four week old female BALB/c mice received 3 i.p. injections of a 100µg mix of Bet v 1-derived peptides or PBS followed by two s.c. sensitisations with 10µg of either rBet v 1 or PBS and 10µg of an unrelated control allergen, rPhl p 5, adsorbed to Al(OH)_3_ at the indicated time points. Thereafter, mice received another injection of the peptide mix or PBS. Time points of injections, bleeding, and sacrifice are indicated. **(B)** Characteristics of the 3 mouse groups.

In one set of experiments mice (n= 6 to 7) received an intraperitoneal (i.p.) injection of a 100µg mix containing 5 long Bet v 1-derived peptides (peptides L1, L2, L3, L4 and L6) and 17 short Bet v 1-derived peptides (peptides S1 to S17) (**Table 1**) on days 2, 4 and 6 (**Figure 1**, Groups 1 and 2). The 100µg peptide mixes were prepared by adding 4.5µg of each peptide from 1mg/ml stock concentrations of reconstituted lyophilized peptides in endotoxin free water and final injection volumes were made up to 150µl per mouse with 50µl of PBS. Group 3 mice received only PBS but not peptides. Group 1 and 3 mice were sensitized with Bet v 1 and all three groups were sensitized to Phl p 5 (**Figure 1**). A second sensitization with Bet v 1 and Phl p 5 was given to group 1 and group 3 mice at week 13 but not to group 2. At week 18, group 1 mice received a second administration of Bet v 1 peptides by the subcutaneous route (**Figure 1**). Serum samples were collected at week 11, 15 and 20. Mice were sacrificed and spleens excised at week 25 for the analysis of CD4^+^ T cell responses. The design of the first tolerance induction experiment allowed to investigate if the administration of peptides would induce allergic sensitization or Bet v 1-specific CD4^+^ T cell or IgG antibody responses because group 2 was not sensitized to Bet v 1. Furthermore, the co-sensitization with Bet v 1 and Phl p 5 of groups 1 and 3 permitted to study the effects of the administration of the Bet v 1 peptides on antibody and T cell responses to the corresponding allergen and to an unrelated allergen.

**Figure 2:**
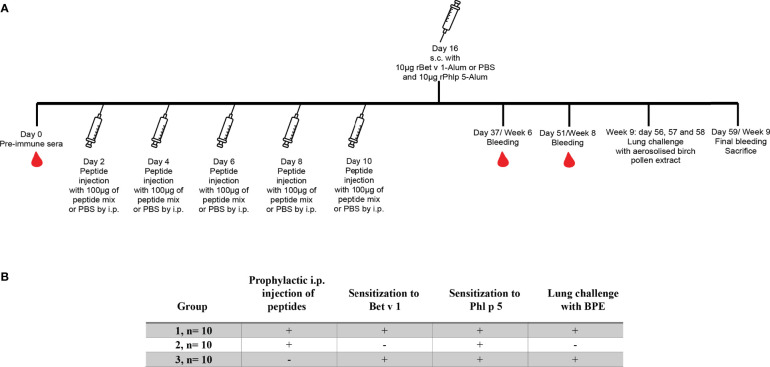
Scheme for the second preventive treatment with more frequent administration of Bet v 1-derived peptides. **(A)** Three to four week old female BALB/c mice received 5 prophylactic i.p. injections of 100µg of a mix of Bet v 1-derived peptides or PBS followed by one s.c. sensitisation with 10µg of either rBet v 1 or PBS together with 10µg of an unrelated control allergen, rPhl p 5, adsorbed to Al(OH)_3_. Thereafter, mice were challenged with either aerosolised birch pollen extract or PBS to assess airway hyper responsiveness for three consecutive days (56, 57 and 58) and then sacrificed on day 59 to examine T cell responses. Time points of prophylactic injections, sensitisation, bleeding, a lung challenge and sacrifice are indicated. **(B)** Characteristics of the 3 mouse groups.

The design of the second set of tolerance experiments was similar to the first experiments with the exception that instead of 3 peptide administrations, 5 were given, a second sensitization and later peptide administration was avoided but a lung challenge was performed (**Figure 2**). Furthermore, the numbers of mice in each group were higher than in the first experiment (i.e., n=10). Respiratory function of mice was measured at days 56, 57 and 58 (week 9) by challenging mice with either PBS or with 0.625mg birch pollen extract dissolved in 125µl of PBS and aerosolized per mouse using Whole Body Plethysmography (Buxco FinePointe, Data Sciences International, USA). Mice were sacrificed and spleens excised at day 59. Serum samples were collected at weeks 6, 8 and 9.

### Assessment of Allergen-Specific CD4^+^ T Cell Proliferation and Cytokine Production

Mouse spleens were removed under aseptic conditions and single cell suspensions were prepared by physical disruption of spleens through 70µM nylon cell strainers (FalconTM, BD Biosciences, New Jersey, USA). Thereafter, isolated splenocytes were labelled with CFSE (Life Technologies, Bleiswijk, Netherlands). Cells were diluted to a final concentration of 2 x 10^5^ cells/well and seeded into 96-well round bottom cell culture plates in triplicates (200µl/well) in the presence or absence of antigens in supplemented RPMI 1640 medium (Gibco, Bleiswijk, The Netherlands), using 5µg/well or 10µg/well of Bet v 1 or Phl p 5, 2.5µg/well or 5µg/well of Bet v 1-derived long peptides L1 to L7, respectively (**Table 1**). Cells were cultured for 5 days and stained with fixable viability dye, eFluorTM 780 (Invitrogen, California, USA), PE/Cy7 anti-mouse CD3 antibody (clone: 17A2, Biolegend) and PE Rat anti-mouse CD4 antibody (Clone: RM4-5, BD Biosciences). Flow cytometry was performed with a FACS Canto flow cytometer (Becton Dickinson) and analysis with the FlowJo software (TreeStar, Ashland, Oregon) (33). The measurement of allergen-specific cytokine production in splenocyte cultures is described in the supplement.

### Measurement of Allergen-Specific Antibodies

Allergen-specific IgE, IgG_1_ and IgG_2a_ antibody levels were measured by ELISA. ELISA plates (Greiner bio-one, Frickenhausen, Germany) were coated in duplicates with 1µg/ml of antigen dissolved in PBS (Bet v 1, Phl p 5, Mal d 1, Cor a 1, Dau c 1, Api g 1, Aln g 1, Bet v 1-derived long peptides L1 to L7) (50µL/well) for five hours at room temperature. Plates were washed twice with PBS 0.05% Tween 20 (200µL/well) and then blocked with 2%BSA PBS 0.05% Tween-20 (100µL/well) at 4°C overnight. Thereafter, sera were added (50µL/well) in duplicates per mouse for incubation at 4°C overnight with the following dilutions: 1:100 for the detection of IgG_1_, 1:10 for the detection of IgE and 1:50 for the detection of IgG_2a_. Plates were then washed five times with PBS 0.05% Tween-20 followed by incubation with a 1:1000 dilution (50µL/well) of purified rat anti-mouse IgE (Clone: R35-72), purified rat anti-mouse IgG_1_ (Clone: A85-1) or purified rat anti-mouse IgG_2a_ (Clone R19-15) (BD Pharmingen, San Diego, CA, USA) at 4°C overnight. Plates were again washed five times as above and then incubated with a 1:1000 dilution (50µL/well) of ECL™ anti-rat IgG, horseradish peroxidase linked whole antibody from goat (Sigma-Aldrich, UK) at 37°C for one hour. Plates were washed five times and colorimetric detection was done with 2,2′-azino-bis 3-ethylbenzothiazoline-6-sulphonic acid (ABTS) (Sigma-Aldrich, St. Louis, Mo, USA) solution in citric acid buffer (50µL/well). Optical densities (OD) were measured using a Tecan infinite F50 instrument (OD at 405nm and reference OD at 492nm). Results are expressed as means of duplicates per mouse with a deviation of less than 10%.

### Degranulation Experiments With Rat Basophil Leukaemia Cells

Rat basophil leukaemia cells (RBL-2H3) grown in supplemented RPMI 1640 medium (Gibco, Bleiswijk, The Netherlands) were seeded at 4 x 10^5^ cells/well into 96-well flat bottom cell culture plate (Corning incorporated, Kennebank, OH) and cultured overnight at 37°C in 5% CO_2_. Cells were then incubated with 1:10 dilutions of a serum pool generated from each mouse-group in triplicates at 37°C and 5% CO_2_ for 2 hours. Thereafter, supernatants were removed and cells were washed twice with Tyrode’s buffer/0.1% BSA (Sigma-Aldrich, UK). IgE-loaded cells were then stimulated with different concentrations of antigens at 37°C for 30 minutes and mediator release was detected in the cell supernatants with the addition of 4-methylumbelliferyl β-D-galactopyranoside (4-MUG, Sigma Aldrich). For determination of 100% mediator release, cells were lysed with 10% v/v Triton X-100 (Merck Millipore, Darmstadt, Germany). The fluorescence of β-hexosaminidase release was measured between wavelengths of 360nm to 465nm using an Infinite 200 PRO microplate reader (Tecan, Maennedorf, Switzerland). The results are calculated as the percentages of total β-hexosaminidase released with the addition of 10% triton.

### Statistical Analysis

Data were analysed using Stata 13.1 (StataCorp, College Station, TX). Peptides were analysed by a general linear model with a log link, comparisons of peptide-induced antibody reactivities and CD4^+^ T cell proliferations against medium control were done by Dunnett’s tests. For prophylactic experiments, data were analysed by a general linear model with a log link, comparing group 1 and 3 by linear contrast. P values of less than 0.05 were considered statistically significant (* P < 0.05; ** P < 0.01; *** P < 0.001).

## Results

### Establishment of a Model for IgE Sensitization to Bet v 1 in BALB/c Mice

**Figure 3:**
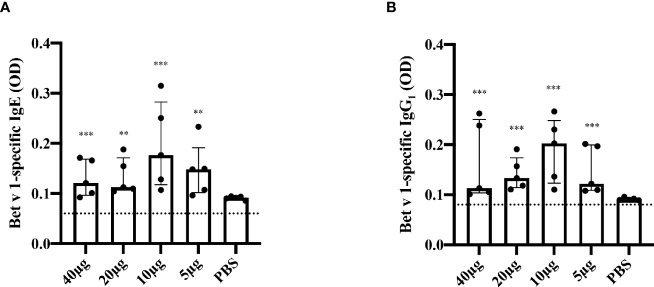
Bet v 1-specific IgE and IgG_1_-antibody responses after single subcutaneous sensitization with different Bet v 1 doses. Shown are serum levels (optical densities, ODs) of Bet v 1-specific IgE **(A)** and Bet v 1-specific IgG_1_ **(B)** as scatter plots with medians and interquartile ranges (*y-axes*) for groups of mice (n = 5) four weeks after one subcutaneous injection of Al(OH)_3_ adsorbed Bet v 1 (40µg, 20µg, 10µg or 5µg) or PBS (*x-axes*). Each data point represents the mean of a duplicate determination from an individual mouse. Data were analyzed by a general linear model with a log link, comparing Bet v 1 sensitized groups to PBS control group by linear contrast. Statistically significant differences between Bet v 1 sensitized groups to PBS control group are indicated (***P < 0.001, **P < 0.01).

In order to establish a model for Bet v 1-specific sensitization, a single injection was selected to determine the effect of one dose on allergen-specific IgE and IgG production. Bet v 1-specific antibody responses could be induced with a single subcutaneous injection of 5μg of Bet v 1. The antibody levels were higher in the group receiving 10μg than in the 5μg group and decreased again when higher Bet v 1-doses were applied (**Figures 3A, B**). Moreover, 10µg dose had consistently (both for IgG_1_ and IgE) the highest average OD values; for IgE this was significantly higher against 20µg (p=0.045) and 40µg (p=0.033). No Bet v 1-specific antibodies were detected in mice immunized only with adjuvant and PBS alone.

### C-Terminal Bet v 1 Peptides Comprise Bet v 1-Specific T Cell Epitopes

**Figure 4:**
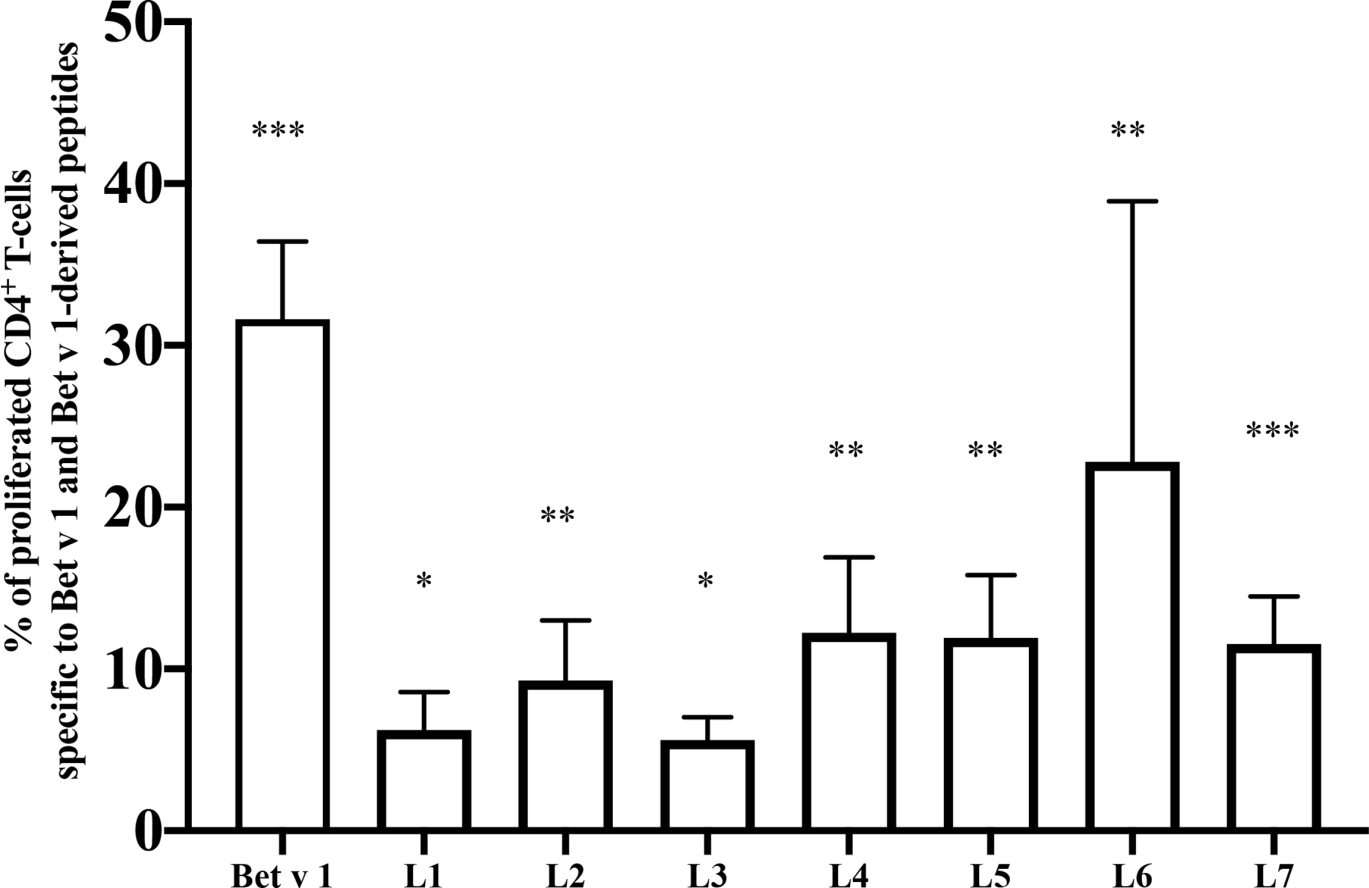
Mapping of T cell epitopes in Bet v 1 sensitized BALB/c mice. Shown are the percentages of proliferated CD4^+^ T cells (y-axis) after subtraction of the medium control specific for Bet v 1 and Bet v 1-derived long peptides (L1-L7) (x-axis). BALB/c mice (n = 7) were sensitized twice subcutaneously with 10µg of Bet v 1 as described in methods. After sacrifice, pooled splenocytes were labelled with CFSE and cultured with 5µg of Bet v 1 and 2.5µg of each of the Bet v 1-derived peptides. T cell proliferation responses were analyzed by flow cytometry 5 days after culture and by gating on live CD3^+^CD4^+^ T cells. Proliferated lymphocytes were assessed by loss of CFSE fluorescence intensity. Bars indicate means of triplicate cultures and standard deviations (SDs). Data were analyzed by a general linear model with a log link, comparisons of Bet v 1 and peptides against medium were done by Dunnett’s tests. Statistically significant differences between medium and Bet v 1, peptides and peptide mix are indicated (***P < 0.001, **P < 0.01, *P < 0.05).

In a first set of experiments, we analyzed T cell proliferations on day 35 after three s.c. injections of 10µg of Bet v 1 every two weeks (days 0, 14 and 28) and found that Bet v 1 and three peptides derived from the Bet v 1 C-terminal portion, peptides L4 (aa 91-126), L6 (aa 119-160) and L7 (aa 130-160) were able to induce specific CD4+ T cell responses above the medium background (data not shown). Bet v 1-specific T cell responses were most pronounced when mice received 2 sensitizations with Bet v 1 10 weeks apart and were sacrificed at week 25 (12 weeks after last sensitization) showing that Bet v 1-specific T cell epitopes in mice include L4, L6 and L7 but are scattered along the sequence with proliferation that were significantly higher than the subtracted medium background (**Figure 4**).

### IgE, IgG_1_ and IgG_2_ Antibodies of Bet v 1-Sensitized Mice Recognize the Same Sequential Bet v 1 Epitopes

**Figure 5:**
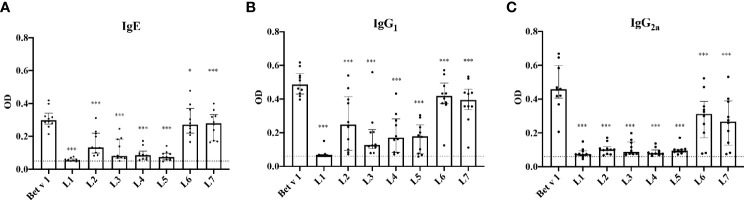
IgE, IgG_1_ and IgG_2a_-recognition of Bet v 1 and Bet v 1 peptides in Bet v 1-sensitized BALB/c mice. Serum levels (optical densities, ODs) (*y-axes*) of Bet v 1- and peptides-specific IgE **(A)**, IgG_1_ **(B)** and IgG_2a_ **(C)** (*x-axes*) are displayed as scatter plots with medians and interquartile ranges. A group of 10 BALB/c mice was sensitized 3 times in intervals of 2 weeks with 10µg of Bet v 1 and ELISAs were performed with sera obtained 4 weeks after the last immunization. Data represent means of duplicate determinations performed for each mouse with a variation of less than 10%. Data were analyzed by a general linear model with a square root link, comparisons of peptides against medium control were done by Dunnett’s test. Statistically significant differences between Bet v 1 and peptides are indicated (***P < 0.001, *P < 0.05).

Next, sera from Bet v 1-sensitized mice were used to identify peptides that react with IgE, IgG_1_ and IgG_2a_ antibodies. In this context it is of note that Bet v 1-allergic patients lack IgE antibodies specific for sequential Bet v 1 epitopes but only recognize the complete folded Bet v 1 molecule (32, 35). We found that IgE antibodies from Bet v 1-sensitized mice recognized not only the complete folded Bet v 1 allergen but also sequential epitopes represented by peptides L2, L6 and L7 (strength of peptide IgE reactivity: peptide L6 = peptide L7 > peptide L2) and also showed some low reactivity with peptides L3, L4 and L5 (**Figure 5A**). Peptides L6 and L7 were significantly higher compared to all peptides except L2. Peptides L6 and L7 form a homogenous subgroup significantly (p<0.05) different from all other peptides for IgG_2a_. For IgE and IgG_1_ L2 joins L6 and L7 as a homogenous subgroup as per Tukey’s HSD test. The same sequential epitopes were recognized with comparable intensity by IgG**_1_** and to a lesser extent by IgG_2a_ antibodies (**Figures 5B, C**).

**Figure 6:**
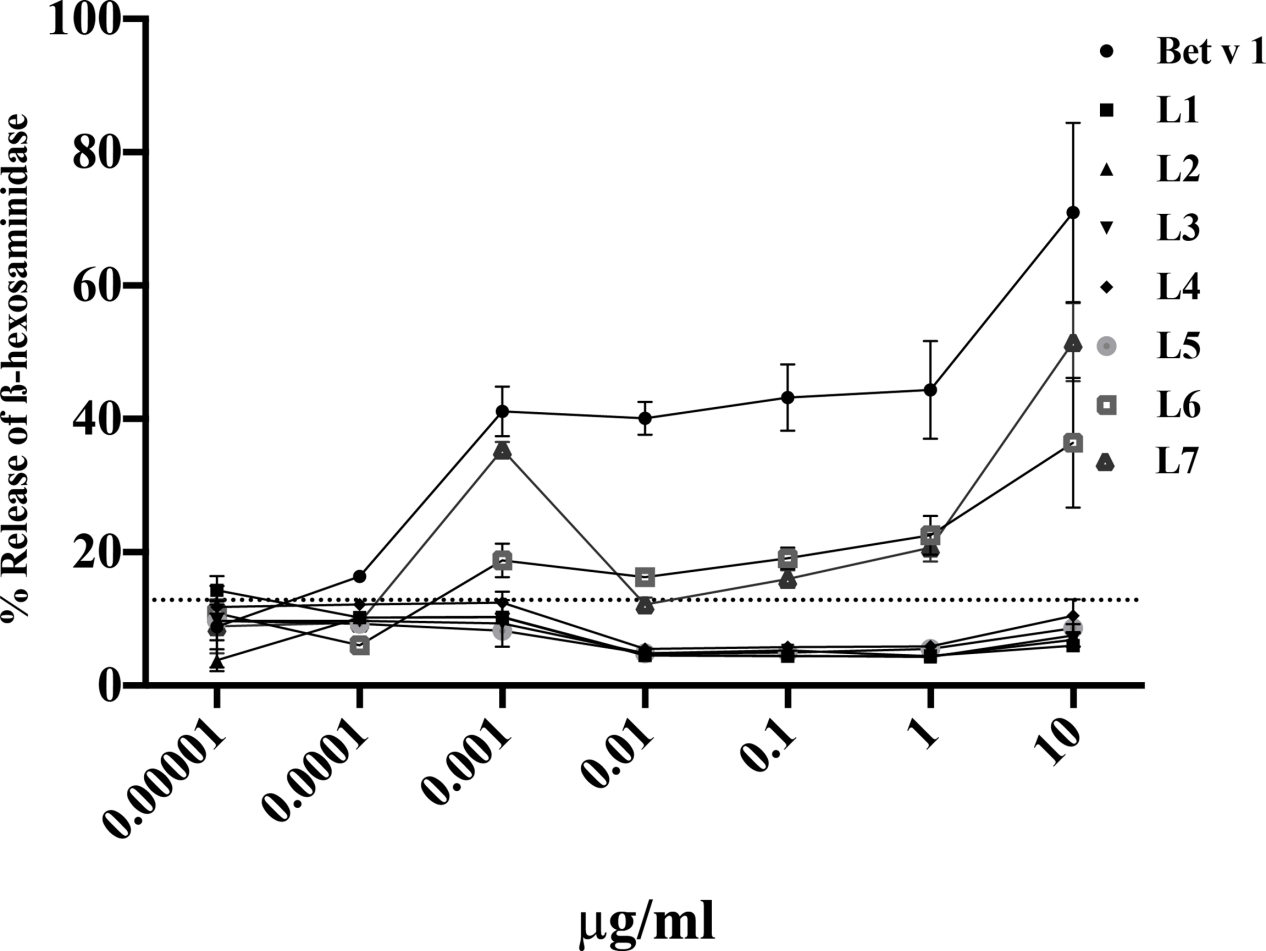
Basophil activation responses with sera from Bet v 1 sensitized mice upon incubation with Bet v 1 and Bet v 1-derived peptides. RBL-2H3 cells were incubated with pooled sera from Bet v 1-sensitised mice (n=10) and degranulation was determined in response to different concentrations (0.00001µg/ml – 10µg/ml) of Bet v 1 or Bet v 1 peptides (L1-L7) (*x-axes*). The releases are shown as mean percentages and standard deviations of total β-hexosaminidase release for triplicate determinations (y-axes). The control measurements with pre-immune sera reveal an upper 2-sigma limit value of 12.83%. All means above this line are significantly different from control measurements.

We also investigated if the peptides can induce specific basophil activation by cross-linking Bet v 1-specific IgE on basophils (**Figure 6**). Bet v 1 induced dose-dependent basophil activation starting already at a concentration of 0.001µg/ml. Specific basophil activation was also obtained with peptides L6 and L7 but an approximately 10,000-fold higher concentration in terms of microgram and more than 40,000-fold higher in terms of molarity was needed to induce comparable basophil activation. Approximately 40% ß-hexosaminidase release was obtained with 0.001µg/ml rBet v 1 whereas 10µg/ml of peptides L6 and L7 were required to achieve a comparable release (**Figure 6**).

### Hierarchy of IgE and IgG Cross-Reactivity in Bet v 1-Sensitized Mice

Bet v 1-allergic patients show IgE cross-reactivity to Bet v 1-related pathogenesis-related proteins (PR10 allergens) in pollen and plant foods (28). IgE antibodies from Bet v 1-sensitized mice showed comparable reactivity to the related pollen allergens from hazel, Cor a 1 and alder, Aln g 1 (**Supplementary Figure 2A**). Lower IgE reactivity was observed with the major apple allergen, Mal d 1 forming an intermediate subset whereas almost no reactivity with Dau c 1 and Api g 1 from carrot and celery, respectively, was found (**Supplementary Figure 2A**). Regarding IgG_1_ strong cross-reactivity with Mal d 1, Cor a 1 and Aln g 1 was found whereas IgG_1_ reactivity to Dau c 1 and Api g 1 was low and they form a homogenous subset significantly different from all other allergens (**Supplementary Figure 2B**). Similar results were obtained for IgG_2a._ Cross-reactivity was most pronounced for Mal d 1, Cor a 1 and Aln g 1 and almost no reactivity to Dau c 1 and Api g 1 was found (**Supplementary Figure 2C**). Thus, for IgG_2a_ reactivity there are three homogenous subsets: Dau c 1 and Api g 1, Mal d 1 and Cor a 1, Aln g 1 and Bet v 1. Subsets significantly differ from each other at p<0.05 as per Tukey’s HSD test.

Aln g 1 induced also strong basophil activation when basophils had been loaded with IgE from Bet v 1-sensitized mice, but Bet v 1 was approximately tenfold more potent in inducing basophil activation (**Supplementary Figure 3**). We noted also basophil activation with the other Bet v 1-related PR10 allergens but only at the highest concentrations tested (i.e., 10µg/ml) (**Supplementary Figure 3**).

The cross-reactivity of IgE and IgG antibodies in Bet v 1-sensitized mice with Bet v 1-related PR10 allergens reflected the extent of sequence identity. **Supplementary Figure 4** shows that the sequences of Aln g 1 (81% sequence identity), Cor a 1 (73%) and Mal d 1 (56%) are most closely related to Bet v 1 whereas the sequence identity of Bet v 1 with Api g 1 (42%) and Dau c 1 (38%) is low.

### Characterization of a Mix of Bet v 1 Peptides Comprising Peptides Capable of Stimulating Mouse and Human CD4^+^ T Cells

Next we were interested to investigate if it was possible to reduce allergic sensitization as well as adaptive immunity to Bet v 1 in terms of IgG and CD4^+^ T cell responses by preventive administration of hypoallergenic T cell-epitope-containing Bet v 1 peptides. Major questions to be addressed in these experiments were i.) if specific tolerance can be induced with a relatively low peptide dose; ii.) if tolerance can be achieved with peptides longer than 30 amino acids without inducing allergic sensitization with the peptides; iii.) if tolerance induction can be achieved with a peptide mix comprising human CD4^+^ T cell epitopes and iv.) if peptide tolerance is specific for the corresponding allergen or if there can be bystander effects.

Testing of the seven long Bet v 1 peptides for their ability to stimulate CD4^+^ T cells revealed that in addition to peptides L6 and L7 containing a previously reported T cell epitope recognized by Bet v 1-sensitized BALB/c mice, peptide L4 contains an additional not yet described CD4^+^ T cell epitope for BALB/c mice (**Figure 4**). In addition, we added a mix of 17 12mer peptides spanning the Bet v 1 C-terminal portion ranging from amino acid positions 85 to 160. This was done to ensure that the newly discovered CD4^+^ T cell-stimulating peptide in peptide L4 and eventual other T cell epitopes of the C-terminus are covered. On the other hand, the mix of short peptides did not contain the previously reported intact BV139 sequence which was part of the long peptides L6 and L7. The peptide mix contained only 4.5µg of each of the peptides and thus the dose of the individual peptides was at least five-fold lower than in earlier tolerance induction experiments carried out with Bet v 1 peptides (31) and more than 50-fold lower than in earlier reports of peptide-induced tolerance (36, 37).

The seven long Bet v 1 peptides (**Table 1**: L1-L7) even covered more completely the Bet v 1 sequence than 6 similar Bet v 1 peptides (**Table 1**: P1-P6) which comprised human Bet v 1-specific CD4^+^ T cell epitopes (32). The latter six peptides P1-P6 were shown to stimulate CD4^+^ T cell responses in blood samples obtained before, during and after the birch pollen season in a population of highly birch pollen-exposed subjects (birch pollen allergic patients: n=6; patients with allergy without birch sensitization: n=4; non-allergic subjects: n=9) from the Moscow region in Russia (**Supplementary Figure 1**). None of the latter mentioned birch pollen allergic patients showed IgE reactivity to the peptides (data not shown).

### Preventive Induction of Bet v 1-Specific Tolerance With a Mix of Bet v 1 Peptides Comprising Peptides Capable of Stimulating Mouse and Human CD4^+^ T Cells

**Figure 7:**
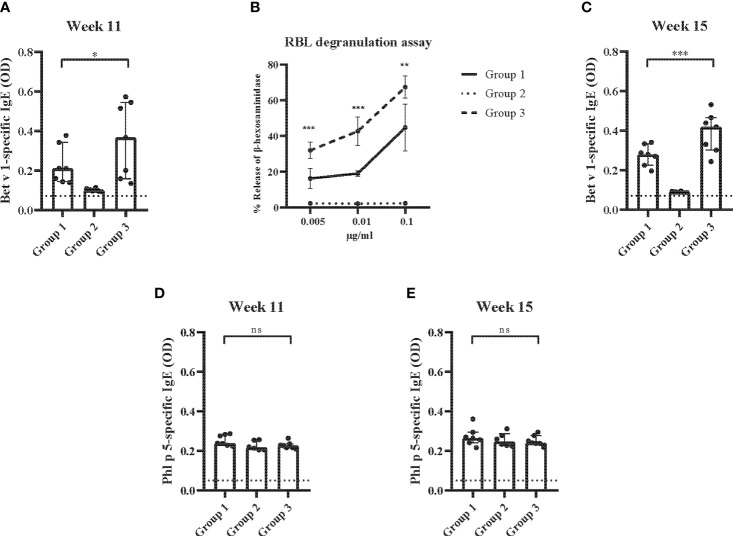
Allergen-specific IgE responses and basophil activation after prophylactic administration of Bet v 1 peptides. **(A)** Bet v 1-specific IgE antibody levels of the three mouse groups at week 11 (*y-axes*: optical density OD levels, group medians with interquartile ranges). **(B)** Bet v 1-specific basophil degranulation (0.005µg/ml, 0.001µg/ml, 0.1µg/ml; *x-axis*) in the three mouse groups at week 11. The percentages of total β-hexosaminidase release are displayed on the y-axis. **(C)** Bet v 1-specific IgE at week 15 (y-axes: optical density, OD levels, group medians with interquartile ranges). Phl p 5-specific IgE antibody levels in the three mouse groups at week 11 **(D)** and at week 15 **(E)** (y-axes: optical density, OD levels, group medians with interquartile ranges). Dot represents means of duplicate determinations in individual mice with less than 10% deviation. Dotted horizontal bars indicate the cut-off levels for the antibody measurements. Data were analyzed by a general linear model with a log link, comparing group 1 and 3 by linear contrast. Statistically significant differences between group 1 and group 3 are indicated (***P < 0.001, **P < 0.01, *P < 0.05). ns, not significant.

In a first set of experiments (**Figure 1**), Bet v 1-specific IgE antibody levels eight weeks after the sensitization (i.e., at week 11) were significantly lower in group 1 which had received preventive administration of Bet v 1 peptides as compared to group 3 which had received only PBS (**Figure 7A**). No Bet v 1-specific IgE was detected in group 2. This group had been sensitized only to Phl p 5 but not to Bet v 1 but had received preventive administration of Bet v 1 peptides indicating that the peptides were non-allergenic since they also failed to prime mice for IgE production against Bet v 1 (**Figure 7A**). An approximately tenfold lower Bet v 1-specific allergic sensitization was found for group 1 mice as compared to group 3 mice in the basophil release experiments at the same time point (**Figure 7B**). Group 2 mice showed no Bet v 1-specific basophil degranulation (**Figure 7B**). Even after a second Bet v 1-specific sensitization at week 13, Bet v 1-specific IgE levels remained significantly lower in group 1 mice as compared to group 3 mice as determined at week 15 (**Figure 7C**). Also, at week 15 no Bet v 1-specific IgE was detected in group 2 mice, again suggesting that peptides were non-allergenic (**Figure 7C**). The specificity of the reduction of Bet v 1-specific allergic sensitization by preventive administration of Bet v 1 peptides was demonstrated by the fact that Phl p 5-specific IgE levels were comparable among the three groups of mice at both, week 11 and at week 15 (**Figures 7D, E**).

**Figure 8:**
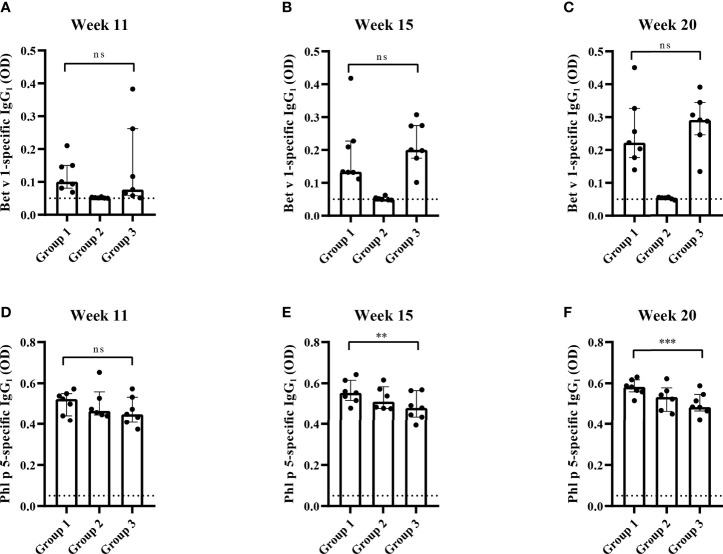
Allergen-specific IgG_1_ responses after prophylactic administration of Bet v 1 peptides. Bet v 1-specific IgG_1_ levels in the three mouse groups at week 11 **(A)**, at week 15 **(B)** and at week 20 **(C)**. Phl p 5-specific IgG1 levels in the three mouse groups at week 11 **(D)**, at week 15 **(E)** and at week 20 **(F)**. Serum levels were measured as duplicates for each mouse with deviations of less than 10% (y-axes: optical density OD levels). Results are displayed as scatter plots with group medians with interquartile ranges. Dotted horizontal bars indicate the cut-off levels for the antibody measurements. Data were analyzed by a general linear model with a log link, comparing group 1 and 3 by linear contrast. Statistically significant differences between group 1 and group 3 are indicated (***P < 0.001, **P < 0.01). ns, not significant.

The analysis of Bet v 1-specific IgG_1_ levels showed that there were no differences between group 1 and group 3 at week 11 whereas at weeks 15 and 20 Bet v 1-specific IgG_1_ levels were lower in group 1 compared to group 3, albeit not reaching statistical significance (**Figures 8A–C**). No Bet v 1-specific IgG_1_ levels were found in group 2 mice at weeks 11, 15 and 20 indicating that the Bet v 1 peptides were non-immunogenic (**Figures 8A–C**).

There was no significant difference between Phl p 5-specific IgG_1_ levels between the three mouse groups at week 11 (**Figure 8D**). At weeks 15 and 20 Phl p 5-specific IgG_1_ levels were even significantly lower in group 3 than in group 1 mice (**Figures 8D–F**).

**Figure 9:**
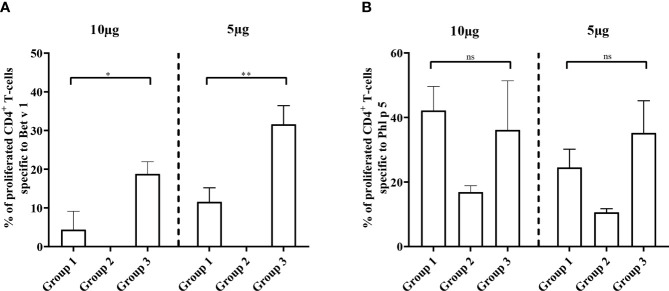
Allergen-specific T cell responses after prophylactic administration of Bet v 1 peptides. Pooled splenocytes from the three mouse groups were labelled with CFSE and cultivated in the presence of 10µg of Bet v 1 or 5µg of Bet v 1 **(A)**, 10µg of Phl p 5 or 5µg Phl p 5 **(B)**. T cell proliferative responses were analyzed by flow cytometry 5 days after initiation of culture and by gating on live CD3^+^CD4^+^ T cells. Proliferated lymphocytes were assessed by loss of CFSE fluorescence intensity. The experiments were performed in triplicates. Shown are the mean percentages of allergen-specific proliferated CD4^+^ T cells after subtraction of the medium controls and SD. Data were analyzed by a general linear model with a log link, comparing group 1 and 3 by linear contrast. Statistically significant differences between group 1 and group 3 are indicated (**P < 0.01, *P < 0.05). ns, not significant.

The reduction of Bet v 1-specific antibody production achieved by preventive administration of Bet v 1 peptides was also reflected at the level of CD4^+^ T cell responses. **Figure 9A** shows that the proliferation of Bet v 1-specific CD4^+^ T cells was significantly lower in group 1 mice as compared to group 3 mice regardless if 5 or 10 µg/ml of Bet v 1 were used for stimulation. However, no proliferation of Bet v 1-specific CD4^+^ T cells was observed in group 2 mice confirming that Bet v 1 peptides despite repeated administrations (three at days 2, 4 and 6) were not immunogenic and did not induce Bet v 1-specific CD4^+^ T cell responses at both concentrations of Bet v 1 used for stimulation of T cells (**Figure 9A**).

There were no significant differences regarding Phl p 5 specific CD4^+^ T cell proliferation between groups 1 and 3 at two different antigen concentrations used for stimulation (**Figure 9B**). The Phl p 5-specific CD4^+^ T cell proliferation in group 2, which had received only one injection with Phl p 5 on day 16, was much lower than that in groups 1 and 3 which had received two injections of Phl p 5, one on day 16 and one at week 13 (**Figures 1**, **9B**).

Furthermore, the analysis of allergen-specific cytokine production in splenocyte cultures showed a significant reduction of Bet v 1-specific IL-4 (5 or 10µg/ml of Bet v 1) and IFN-γ levels (10µg/ml of Bet v 1) in Bet v 1-peptide-tolerized group 1 mice compared to group 3 mice but not for the control allergen Phl p 5 (**Supplementary Figure 5**). There were no significant differences regarding Bet v 1-induced IL-5, IL-13, IL-10 and TGF-β levels in splenocyte cultures stimulated with 5 or 10µg/ml of Bet v 1 between group 1 and group 3 mice (**Supplementary Figure 5**). Similar to the absence of Bet v 1-specific CD4^+^ T cell proliferation observed in group 2 mice, there was no relevant induction of Bet v 1 specific IL-4, IL-5 and IL-13 in group 2 mice (**Supplementary Figure 5**). With few exceptions (i.e., IL-5 and IL-10) cytokine responses to both concentration of the control allergen Phl p 5 were similar in all three mouse groups (**Supplementary Figure 5**).

**Figure 10:**
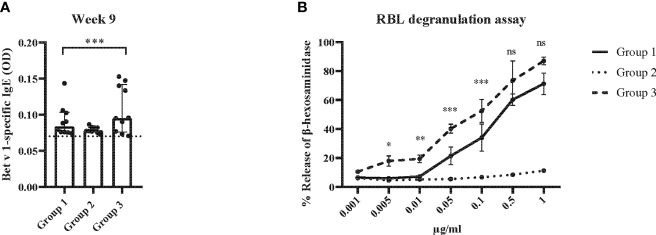
Allergen-specific IgE responses and basophil activation after five prophylactic administrations of Bet v 1 peptides. **(A)** Bet v 1-specific IgE antibody levels of the three mouse groups at week 9 ( (y-axes: optical density, OD levels, group medians with interquartile ranges). Dots represent means of duplicate determinations in individual mice with less than 10% deviation. Dotted horizontal bar indicates the cut-off levels for the antibody measurements. **(B)** Bet v 1-specific basophil degranulation (0.001µg/ml-1µg/ml; x-axis) in the three mouse groups at week 9. The percentages of total β-hexosaminidase release are displayed on the y-axis.. Data were analyzed by a general linear model with a log link, comparing group 1 and 3 by linear contrast. Statistically significant differences between group 1 and group 3 are indicated (***P < 0.001, **P < 0.01, *P < 0.05). ns, not significant.

**Figure 11:**
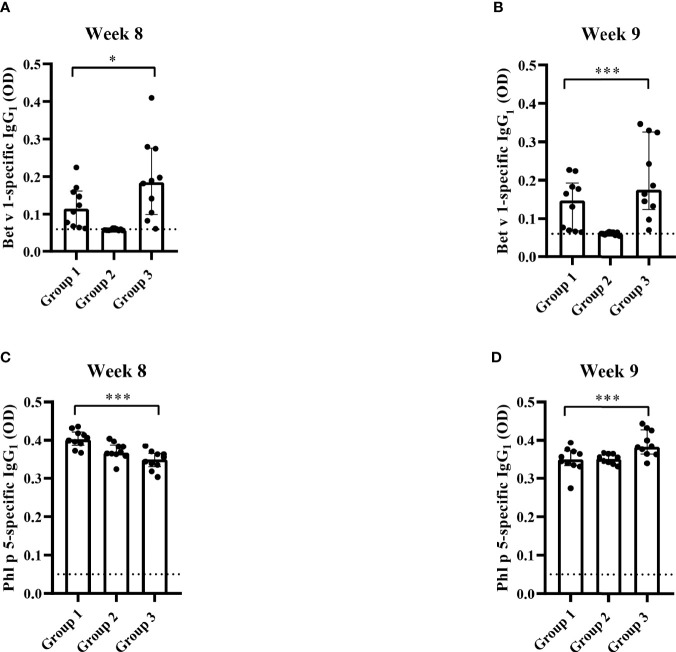
Allergen-specific IgG_1_ responses after five prophylactic administrations of Bet v 1 peptides. Bet v 1-specific IgG1 levels in the three mouse groups at week 8 **(A)** and at week 9 **(B)**. Phl p 5-specific IgG1 levels in the three mouse groups at week 8 **(C)** and at week 9 **(D)**. Serum levels were measured as duplicates for each mouse with a deviation of less than 10% (y-axes: optical density OD levels). Results are displayed as scatter plots with group medians with interquartile ranges. Dotted horizontal bars indicate the cut-off levels for the antibody measurements. Data were analyzed by a general linear model with a log link, comparing group 1 and 3 by linear contrast. Statistically significant differences between group 1 and group 3 are indicated (***P < 0.001, *P < 0.05).

**Figure 12:**
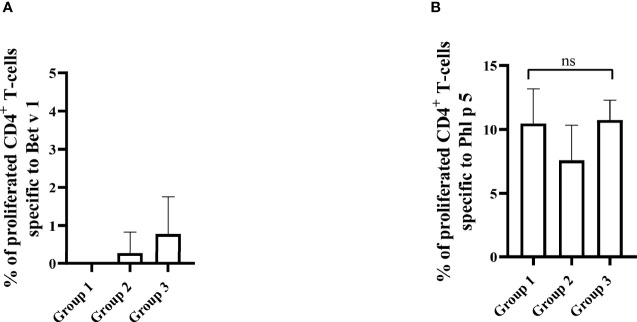
Allergen-specific T cell responses after five prophylactic administrations of Bet v 1 peptides. Pooled splenocytes from the three mouse groups were labelled with CFSE and cultivated in the presence of 10µg of Bet v 1 **(A)** or 10µg of Phl p 5 **(B)**. T cell proliferative responses were analyzed by flow cytometry 5 days after culture and by gating on live CD3^+^CD4^+^ T cells. Proliferated lymphocytes were assessed by loss of CFSE fluorescence intensity. The experiments were performed in triplicates. Shown are the mean percentages of allergen-specific proliferated CD4^+^ T cells after subtraction of the medium controls and SD. Data were analyzed by a general linear model with a log link, comparing group 1 and 3 by linear contrast. Statistically significant differences between group 1 and group 3 are indicated. ns, not significant.

The results obtained in the second set of experiments (**Figure 2**) were almost identical to the ones obtained in the first set of experiments. Preventive administration of Bet v 1 peptides yielded significantly lower Bet v 1-specific IgE levels in group 1 as compared to group 3 (**Figure 10A**). Again, no induction of Bet v 1-specific IgE and basophil sensitivity was noted in group 2 which had been treated only with peptides (**Figure 10A**). Group 1 mice were approximately fivefold less sensitive to Bet v 1 in basophil degranulation experiments (**Figure 10B**). Bet v 1-specific IgG_1_ antibody levels were significantly lower in group 1 mice compared to group 3 at weeks 8 and 9 (**Figures 11A, B**) whereas Phl p 5-specific IgG_1_ levels were higher in group 1 than in group 3 at week 8 (**Figure 11C**) and lower at week 9 (**Figure 11D**). No Bet v 1-specific CD4^+^ T cells were detected in group 1 mice whereas they were clearly detectable in group 3 mice (**Figure 12A**). No significant difference regarding Phl p 5-specific CD4^+^ T cells were noted between groups 1, group 2 and group 3 (**Figure 12B**).

The single sensitization to Bet v 1 did not induce any relevant airway symptoms in mice when exposed to inhalation of birch pollen extract as compared to inhalation with saline alone (**Supplementary Figure 6**). Therefore, it was not possible to observe any effects of peptide treatment on lung function.

## Discussion

The goal of our study was to develop in a murine model an allergen-specific prophylactic treatment approach based on the administration of T cell epitope-containing non-allergenic peptides in early life which could be eventually translated into clinical application. Several hurdles for the induction of “neonatal tolerance induction” for allergy prevention by T cell epitope-containing peptides need to be overcome. First of all, it is necessary to demonstrate that early life administration of the peptides does not induce allergic sensitization and it is important to use a mix of peptides comprising the T cell epitope binding (MHC) and recognition (TCR) repertoire of humans. In this context we noted some fundamental differences between the Bet v 1-specific allergic immune response in birch pollen allergic patients and in Bet v 1-sensitized mice. In contrast to birch pollen allergic patients who recognize mainly conformational IgE epitopes on Bet v 1 but lack IgE reactivity to unfolded Bet v 1 fragments or Bet v 1 peptides (32, 35), sensitized mice showed IgE reactivity to several Bet v 1 peptides, i.e., peptides L2-L7, but not to the N-terminal part of the molecule covered by peptide L1 and identified the C-terminal peptides L6 and L7 as major IgE-reactive epitope. Unlike allergic humans, whose IgE and IgG antibodies react with different allergens and allergen epitopes which is indicative of a non-sequential class-switch mechanism (24, 38, 39) we demonstrate that Bet v 1-sensitized mice recognized the same peptides with their IgE, IgG_1_ and IgG_2_ antibodies. This is in agreement with earlier studies suggesting that a sequential class-switch path occurs in sensitized mice (40, 41). Interestingly, we found that a lower dose of Bet v 1 (i.e., 10 µg) was more effective in inducing IgE sensitization as compared to higher doses but this effect was not as pronounced as reported for other allergens (42, 43).

The mapping of CD4^+^ T cell epitopes identified a previously reported dominant T cell epitope BV139 (31) represented in peptides L6 and L7 and a hitherto unknown additional mouse T cell epitope located in peptide L4. In fact, a dominant T cell epitope located at the C-terminus has been described in human Bet v 1 allergic patients but unlike in mice, humans recognize several T cell epitopes in the Bet v 1 allergen which were distributed over the complete molecule (44). In order to represent the human T cell epitope repertoire in our tolerance induction experiments we chose the approach of using relatively long peptides (i.e., longer than 30 amino acids) which has been suggested earlier by von Garnier et al., for the major bee venom allergen, phospholipase A2 (Api m 1) (21). The concept of using long T cell epitope-containing peptides is thought to cover the T cell epitope repertoire of humans more reliably than by using short peptides but bears the risk that the administration of long peptides may induce allergic sensitization. The inclusion of the mouse group 2, which only received the preventive administration of the mix of Bet v 1 peptides without sensitization to Bet v,1 clearly demonstrated that the Bet v 1 peptides neither induced allergic sensitization, nor did they induce Bet v 1-specific IgG antibodies, specific T cell or cytokine responses. This aspect has not been investigated in the study by von Garnier et al. (21), and in the study by Bauer et al. (31), which has investigated the tolerogenic effect of preventive injection of a short immune-dominant Bet v 1 peptide. Like in the study by von Garnier et al., we found that the preventive administration of the mix of Bet v 1 peptides reduced Bet v 1-specific IgE production and IgE-mediated allergic reactions accompanied by reduced T cell proliferation but in addition Bet v 1-specific IgG_1_ responses were reduced in the peptide-treated mice. By contrast, in the study by von Garnier et al., peptide pretreatment enhanced allergen-specific IgG_2a_ responses and did not reduce allergen-specific IgG_1_ responses. This is quite interesting because von Garnier et al., used longer peptides (i.e., 44-60mers) whereas we used peptides with a length of less than 43 amino acids. The fact, that peptides longer than 44 amino acids as well as recombinant Bet v 1 fragments comprising more than 70 amino acids induced IgE and IgG antibodies when injected in humans indicates that in addition to other factors (e.g., dose, use of adjuvants), the length of the “long peptide” determines whether they induce an active immune response characterized by IgE, IgG and T cell responses or immunological tolerance characterized by lack of IgE, IgG and T cell responses (45–47).

In principle, there seem to be at least two possibilities for the allergen-specific reduction/prevention of allergic sensitization. One possibility is to establish an allergen-specific IgG response before sensitization takes place, which is suggested to prevent allergic sensitization by the “blocking activity” of allergen-specific IgG (48, 49). For this purpose, allergen-derived peptides of a similar length as used in our study can be bound to carrier molecules to render them immunogenic. For this approach peptides derived from the IgE binding sites of the allergen are used to focus blocking IgG antibodies towards the IgE epitopes (50).

The other peptide-based approach is based on non-immunogenic T cell epitope-containing peptides for the induction of immunological tolerance reducing allergen-specific T cell and antibody responses. In our study we avoided immunogenicity in order not to induce allergenic sensitization and hence peptides were not bound to a carrier molecule and no adjuvant was used. Furthermore, we included T cell epitope-containing peptides in the tolerogenic peptide mix to induce T cell tolerance. In this context it should be mentioned that certain peptides included in our peptide mix contained also murine B cell epitopes but allergic patients do not show IgE reactivity to the peptides (32, 35).

In certain approaches both mechanisms may be operative (51–53).

The mechanism underlying our approach is clearly induction of immunological tolerance because the administration of the peptides did not induce any detectable adaptive immunity. Measurement of cytokine responses in cultured splenocytes obtained from the three mouse groups suggests that tolerance induced in our model might be attributed to clonal anergy/inactivation of Bet v 1-specific Th2 cells producing IL-4 and, to some extent also of IFN-γ-producing Th1 cells, rather than by immunomodulation towards Th1 or regulation by cells producing IL-10 or other tolerogenic cytokines. Of note we were able to achieve the tolerogenic effect in a reproducible manner with a mix of allergen-derived peptides comprising the human T cell epitope repertoire of Bet v 1. Importantly, we also could show that tolerance induction was possible with considerably lower peptide doses as compared to earlier studies (21, 29–31). Another aspect which discriminates our study from earlier work is that we investigated tolerance induction in a model of allergic co-sensitization to two major immunologically distinct allergens. This is a relevant aspect because allergic patients are usually sensitized to several different allergens and allergic sensitization mostly starts in early childhood towards several unrelated allergens. In this context, it is of note that it has been found in a murine model of co-sensitization that sensitization to one allergen can be modulated by sensitization to an immunologically unrelated allergen (43). We have used co-sensitization to Phl p 5, an allergen which is immunologically unrelated to Bet v 1 in all groups of mice to study the specificity of tolerance induction by Bet v 1 peptides for Bet v 1 and thus can rule out that tolerance induction with Bet v 1 peptides has bystander effects on the immune response towards an unrelated allergen. In this context, it may be interesting to study also other unrelated allergens which may have effects on the innate immune system such as the house dust mite allergen, Der p 1. It will therefore be necessary to define the peptides for the clinically relevant allergens in the allergen sources important for allergic sensitization in different countries and populations. However, this should become possible even for complex allergen sources by using peptides of a length as defined in our study (24).

However, further limitations of our study should be mentioned. The availability of a Bet v 1-humanized mouse which unfortunately is not yet available, would have eventually facilitated our study and the i.p. route of administration will not be possible in infants. We have used this route of application to make sure that the full dose of peptides becomes systemic and to avoid immunogenicity as it may occur during i.m., s.c. or intradermal application. In fact, other more acceptable routes such as subcutaneous or intravenous administration can be used in clinical trials to achieve systemic peptide administration but these were not possible in small mice because of the applied volume. Therefore, other feasible routes of administration such as the s.c. and especially oral administration of peptides need to be developed for clinical application. Regarding the oral route of administration it will be important to develop application forms which allow the systemic uptake of peptides from the gut. Another limitation of our study is that although an approximately tenfold reduction of sensitivity as demonstrated in the basophil activation experiments (**Figure 7B**) was obtained which compares to the effects of allergen-specific immunotherapy (54, 55), the efficacy of treatment must be further improved for example by working on the dosing of peptides. Nevertheless, evidence for the clinical relevance of our results might be obtained by comparing the results from our immunological findings with those obtained in a landmark clinical study investigating tolerance induction in infants at risk for peanut allergy which were published by Du Toit G et al. (11). The authors of this study reported a statistically significant clinical result showing that peanut allergy at 72 months was significantly more prevalent among participants in the peanut avoidance group than among those in the peanut consumption group (18.6% *versus* 4.8%, p<0.001). However, the authors did not find significant differences regarding specific IgE levels to the major peanut allergen Ara h 2 or peanut allergen extracts and they also did not find significant differences regarding peanut allergen-specific skin test results. By contrast, we found that our intervention (i.e., administration of Bet v 1 peptides) resulted in significantly lower Bet v 1-specific IgE levels (**Figures 7A, C**) and a significant and tenfold lower Bet v 1-specific basophil activation, an *in vitro* surrogate for skin tests results, in the peptide-treated *versus* the non-treated mice. The fact that prophylactic administration of allergen peptides had much stronger effects on key immunological parameters (allergen-specific IgE levels and allergen-induced effector cell activation) than peanut consumption would suggest that one may expect clinical relevance for prophylactic peptide administration but of course clinical studies need to be done to study this.

In summary, we demonstrated that it is possible to reduce allergic sensitization to the major birch pollen allergen by preventive administration of a low-dose mix of tolerogenic and non-allergenic Bet v 1 peptides even in a very harsh model of allergic sensitization based on subcutaneous injection of aluminum-hydroxide-adsorbed Bet v 1. Our study may be considered as an early first step towards the translation of preventive approaches based on T cell-epitope containing non-allergenic peptides into clinical studies. There are several additional questions such as dose, route, time window and duration of administration which need to be investigated but we have shown here that the defined Bet v 1 peptides have tolerogenic activity and lack allergenicity.

## Data Availability Statement

The original contributions presented in the study are included in the article/**Supplementary Material**. Further inquiries can be directed to the corresponding author.

## Ethics Statement

The animal study was reviewed and approved by Animal Ethics Committee of the Medical University of Vienna and the Austrian Federal Ministry of Science, Research and Economy BMWFW (66.009/0431-V/3b/2019).

## Author Contributions

OA contributed to design of the study, performed experiments, interpreted the findings, and wrote the manuscript. RC and RV designed the study and contributed to the interpretation of the findings, writing and revising the manuscript. H-JH, BL, MF-T, SV, NC, JE-D, VN-L, AP, AN, MKh, BK, PT, and WP helped in performing experiments, providing materials, read and revised the manuscript. MKu contributed by performing statistical analysis and participated in the interpretation of the findings, read and revised the manuscript. All authors contributed to the article and approved the submitted version.

## Funding

This work was supported by the FWF-funded MCCA PhD-program (W1248-B30), by the Country of Lower Austria (Danube-ARC) and by a Megagrant of the Government of the Russian Federation, grant No 14.W03.31.0024

## Conflict of Interest

RV has received research grants from Viravaxx, Vienna, Austria, HVD Life Sciences, Vienna, Austria, WORG Pharmaceuticals, Hangzhou, China and the country of Lower Austria. He serves as a consultant for Viravaxx, Vienna, Austria and for WORG Pharmaceuticals, Hangzhou, China.

The remaining authors declare that the research was conducted in the absence of any commercial or financial relationships that could be construed as a potential conflict of interest.

## Publisher’s Note

All claims expressed in this article are solely those of the authors and do not necessarily represent those of their affiliated organizations, or those of the publisher, the editors and the reviewers. Any product that may be evaluated in this article, or claim that may be made by its manufacturer, is not guaranteed or endorsed by the publisher.
