# Supplementary material for: Preventive Administration of Non-Allergenic Bet v 1 Peptides Reduces Allergic Sensitization to Major Birch Pollen Allergen, Bet v 1
Source: Front Immunol. 2021 Oct 26;12:744544. doi: 10.3389/fimmu.2021.744544 (PMC8594376; doi:10.3389/fimmu.2021.744544)
Supplement: Supplementary file 1 [file DataSheet_1.docx]

Supplementary Materials

**Supplementary materials and methods**

1. **Sequence alignment**

Multiple sequence alignments of Bet v 1 (Uniprot ID: P15494), Mal d 1 (Uniprot ID: P43211), Cor a 1 (Uniprot ID: Q08407) , Dau c 1 (Uniprot ID: O04298), Api g 1 (Uniprot ID: P49372) and Aln g 1 (Uniprot ID: P38948) was performed using National Center for Biotechnology information, NCBI Blast tool (<https://blast.ncbi.nlm.nih.gov/Blast.cgi>). Then sequences were adjusted and identities and gaps inserted by hand.

1. **Measurement of allergen-specific antibodies**

Allergen-specific IgE, IgG_1_ and IgG_2a_ were measured by ELISA. ELISA plates (Greiner bio-one, Frickenhausen, Germany) were coated in duplicates with 1µg/ml of antigen (Bet v 1, Phl p 5, Mal d 1, Cor a 1, Dau c 1, Api g 1 and Aln g 1) (50µL/well) for five hours at room temperature. Plates were washed twice with PBS, 0.05% Tween 20 (200µL/well) and then blocked with 2%BSA PBS, 0.05% Tween 20 (100µL/well) overnight at 4^o^C. Sera were added (50µL/well) thereafter in duplicates per mouse for overnight incubation at 4^o^C with the following dilutions: 1:100 for the detection of IgG_1_, 1:10 for the detection of IgE and 1:50 for the detection of IgG_2a_ diluted in buffer with 1 part 2%BSA PBS, 0.05% Tween 20 and 3 parts PBS, 0.05% Tween 20. Plates were then washed five times with PBS, 0.05% Tween 20 followed by incubation with a 1:1000 diluted (50 µL/well) purified rat anti-mouse IgE (Clone: R35-72), purified rat anti-mouse IgG_1_ (Clone: A85-1) or purified rat anti-mouse IgG_2a_ (Clone R19-15) (BD Pharmingen, San Diego, CA, USA). Plates were again washed five times as above and then incubated with a 1:1000 diluted (50µL/well) anti-rat IgG, horseradish peroxidase linked whole antibody from goat (Sigma-Aldrich, UK) for one hour at 37^o^C. Plates were then washed five times and colorimetric detection was done with 2,2′-azino-bis 3-ethylbenzothiazoline-6-sulphonic acid (ABTS) (Sigma-Aldrich, St. Louis, Mo, USA) solution in citric acid buffer (50µL/well). Optical densities (OD) were measured using a Tecan infinite F50 ELISA reader (OD at 405nm and reference OD at 492nm). Results are expressed as means of duplicates per mouse with a deviation of less than 10%.

1. **Degranulation experiments with rat basophil leukemia cells**

Rat basophil leukemia cell line RBL-2H3 were seeded at 4 x 10^5^ cells per well into 96 well flat bottom cell culture plate (Corning incorporated, Kennebank, USA) in RPMI 1640 medium (Gibco, Bleiswijk, The Netherlands) (500ml of medium supplemented with 50µM β-Mercapethanol, 2mM Sodium pyruvate, 10µM Hepes buffer, 10ml L-Glutamine, 5ml Pen-strep and 50ml FBS) and cultured overnight at 37^o^C in 5% CO_2_ overnight. Cells were then incubated with 1:10 dilutions in supernatant of sera per mouse-group in triplicates for 2 hours at 37°C and 5% CO_2_. Thereafter, supernatants were removed and cells were washed twice with Tyrode’s buffer/0.1% BSA (Sigma-Aldrich, UK). IgE-loaded cells were then stimulated with different concentrations of the antigens (0.0001 to 10µg/ml) (rBet v 1, rPhl p 5, rMal d 1, rCor a 1, rDau c 1, rApi g 1 and rAln g 1) for 30 min at 37°C and mediator release was detected in the cell supernatants with the addition of 4-methylumbelliferyl β-D-galactopyranoside (4-MUG, Sigma Aldrich). For determination of 100% mediator release (i.e., total release), cells were lysed with 10% v/v Triton X-100 (Merck Millipore, Darmstadt, Germany). The fluorescence of beta-hexosaminidase release was measured between wavelengths of 360nm to 465nm using an Infinite 200 PRO microplate reader (Tecan, Maennedorf, Switzerland). The results were calculated as the percentage of total Beta-hexosaminidase release.

1. **Measurement of allergen-specific cytokine production**

Splenocytes from the three mouse groups were cultured for 5 days in the presence or absence of antigens in supplemented RPMI 1640 medium (Gibco, Bleiswijk, The Netherlands), using 5µg/well or 10µg/well of Bet v 1 or Phl p 5 as described for T cell proliferation experiments. After 5 days of culture supernatants were collected and frozen at -70°C for the analysis of IL-4, IL-5, IL-13, IFN-γ, IL-10 and TGF-β by Luminex technology (Bio-Plex mouse cytokine assay, Bio-Rad) according to the manufacturer’s instructions.

1. **Measurement of airway responsiveness**

On days 56, 57 and 58, respiratory function (Penh) was measured using unrestrained whole body plethysmography (Buxco FinePointe Whole Body, Data Sciences International, USA). Basal readings with PBS only were collected from all mice and averaged for a two minute period. Thereafter, aerosolized PBS or 0.625mg of birch pollen extract in a total volume of 125μl per mouse was applied and Penh were recorded and averaged over a two minute period after nebulization.

**Figure legends**

**Supplementary Figure 1.** Percentages of birch pollen allergic patients (BPA, n=6), subjects with other allergies (n=4) non-allergic subjects (non-allergic n=9) (y-axes) showing specific CD4^+^ T cell responses to different concentrations (1.2nM, 12 nM) of Bet v 1, individual peptides (P1-P6) or to at least one of the 6 peptides (Psum) (x-axes) in spring, summer and autumn in a birch pollen exposed region.

**Supplementary Figure 2.** Cross-reactivity of IgE and IgG antibodies of Bet v 1-sensitised mice with Bet v 1-related PR10 allergens. Shown are serum levels (OD) of specific-IgE (A), IgG_1_ (B) and IgG_2a_ (C) for each mouse of the group (n=10) as scatter plots with medians and interquartile ranges (y-axes).Data represent the means of duplicates for each mouse with a deviation of less than 10%. Horizontal dotted lines indicate the cut-off levels.

**Supplementary Figure 3.** Induction of basophil activation by Bet v 1- and Bet v 1-related PR10 allergens. RBL-2H3 cells were loaded with with pooled serum IgE from Bet v 1-sensitised mice (n=10) and degranulation was induced by adding different concentrations of allergens (0.0001µg/ml – 10µg/ml) (x-axis). The releases are shown as percentages of total β-hexosaminidase calculated from triplicates with SDs (y-axis).

**Supplementary Figure 4.** Multiple sequence alignment of birch pollen allergen of Bet v 1 with Bet v 1-related PR10 proteins Bet v 1-derived peptides are boxed in the sequences (peptide L1, blue; peptide L2, yellow; peptide L3, green; peptide L4, orange; peptide L5, purple; peptide L6, red; peptide L7, light blue; BV139, black). Points indicate identical amino acids and gaps are shown by dashes. The percentages (%) of amino acid sequence identities of each PR10 allergen with Bet v 1 are indicated in the right margin.

**Supplementary Figure 5.** Allergen-specific cytokine responses. Shown are the means and SDs of cytokine levels (y-axes: IL-4, IL-5, IL-13, IFN-γ, IL-10 and TGF-β, pg/ml) measured in triplicate cultures after stimulation with 10µg or 5µg of Bet v 1 (left panel) or 10µg or 5µg of Phl p 5 (right panel) for each mouse group. Results are means of triplicate values after subtraction of the medium controls. Data were analyzed by a general linear model with a log link, comparing group 1 and 3 by linear contrast. Statistically significant differences between group 1 and group 3 are indicated (** P< 0.01, * P< 0.05). ns, not significant.

**Supplementary Figure 6.** Average airway function measurements with PBS and birch pollen extract. Shown are average Penh readings for basal reading with PBS (left panel), and the average Penh readings in response to extract containing either birch pollen or PBS only (right panel) for each mouse of the group (n=10) as scatter plots with medians and interquartile ranges (y-axes). Data represent the mean readings for each mouse from a two minute recording period after nebulization.
