# Supplementary figures and images for: Preventive Administration of Non-Allergenic Bet v 1 Peptides Reduces Allergic Sensitization to Major Birch Pollen Allergen, Bet v 1
Source: Front Immunol. 2021 Oct 26;12:744544. doi: 10.3389/fimmu.2021.744544 (PMC8594376; doi:10.3389/fimmu.2021.744544)

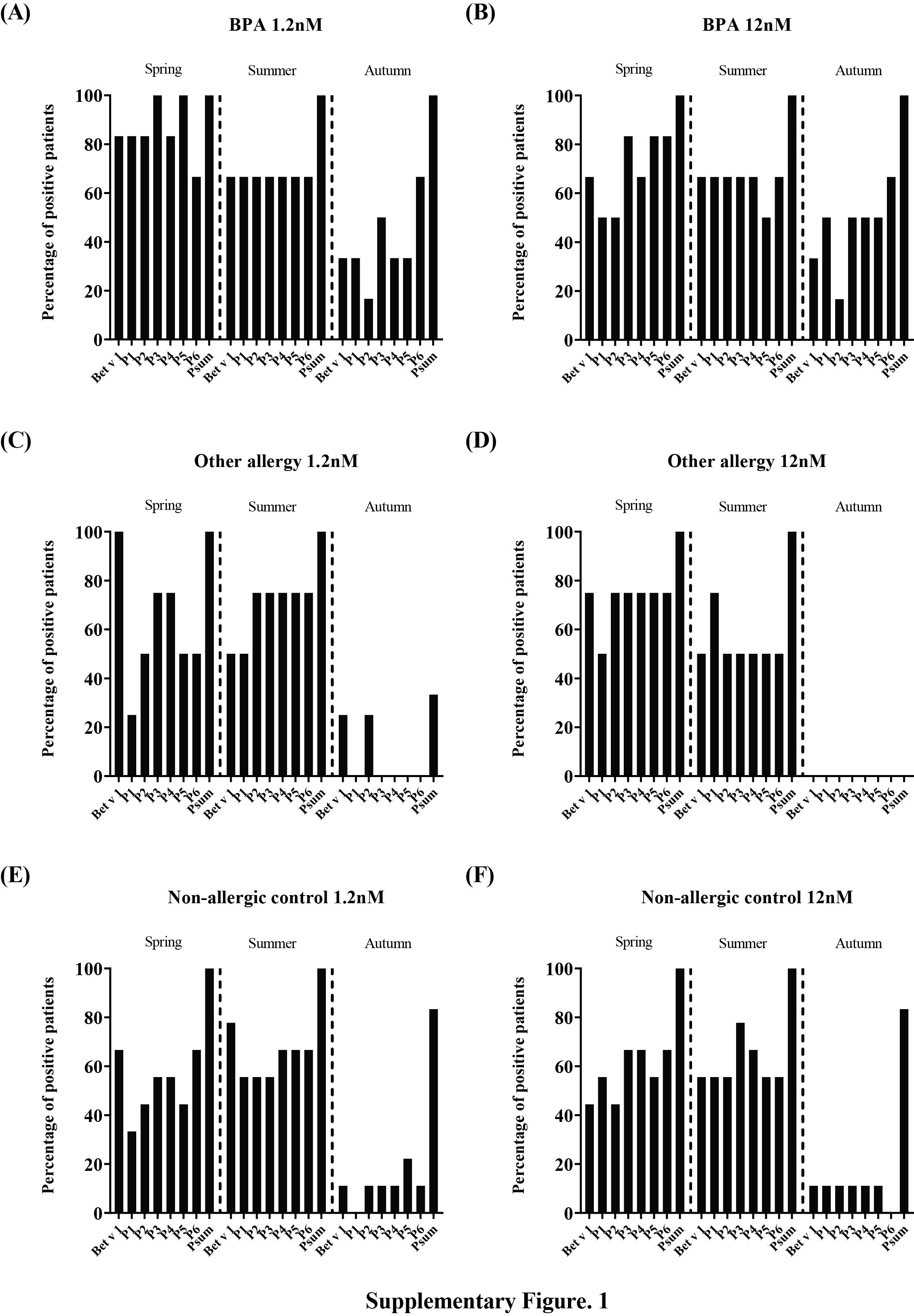

Supplement: Supplementary file 2 [file Image_1.tif]

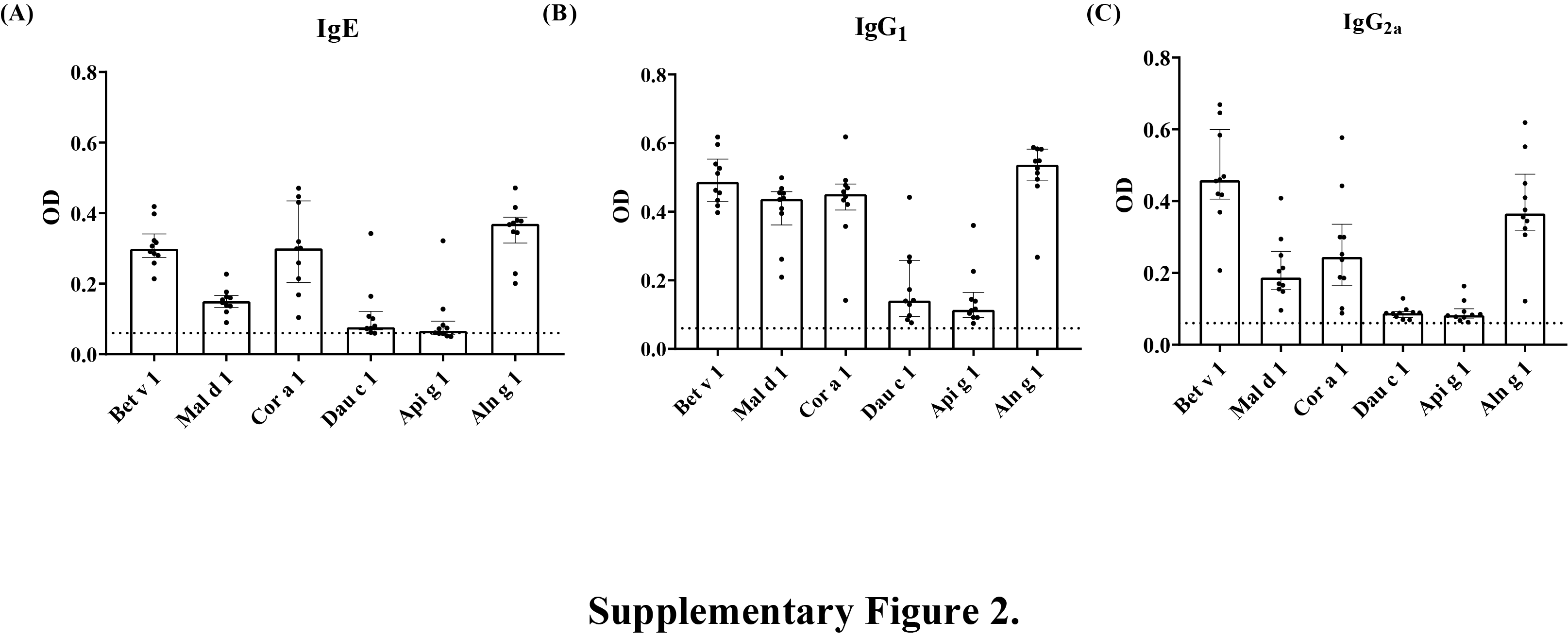

Supplement: Supplementary file 3 [file Image_2.tif]

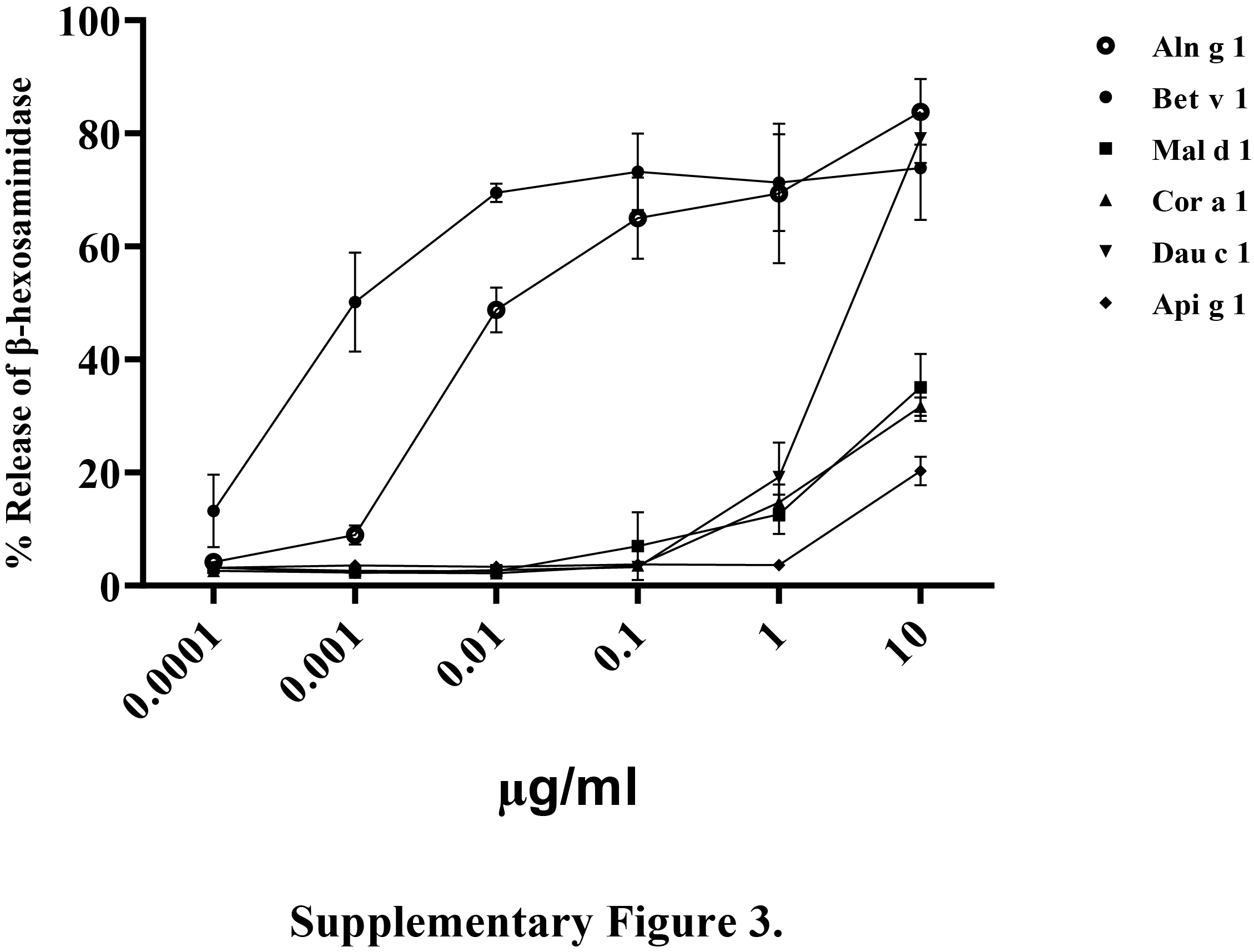

Supplement: Supplementary file 4 [file Image_3.tif]

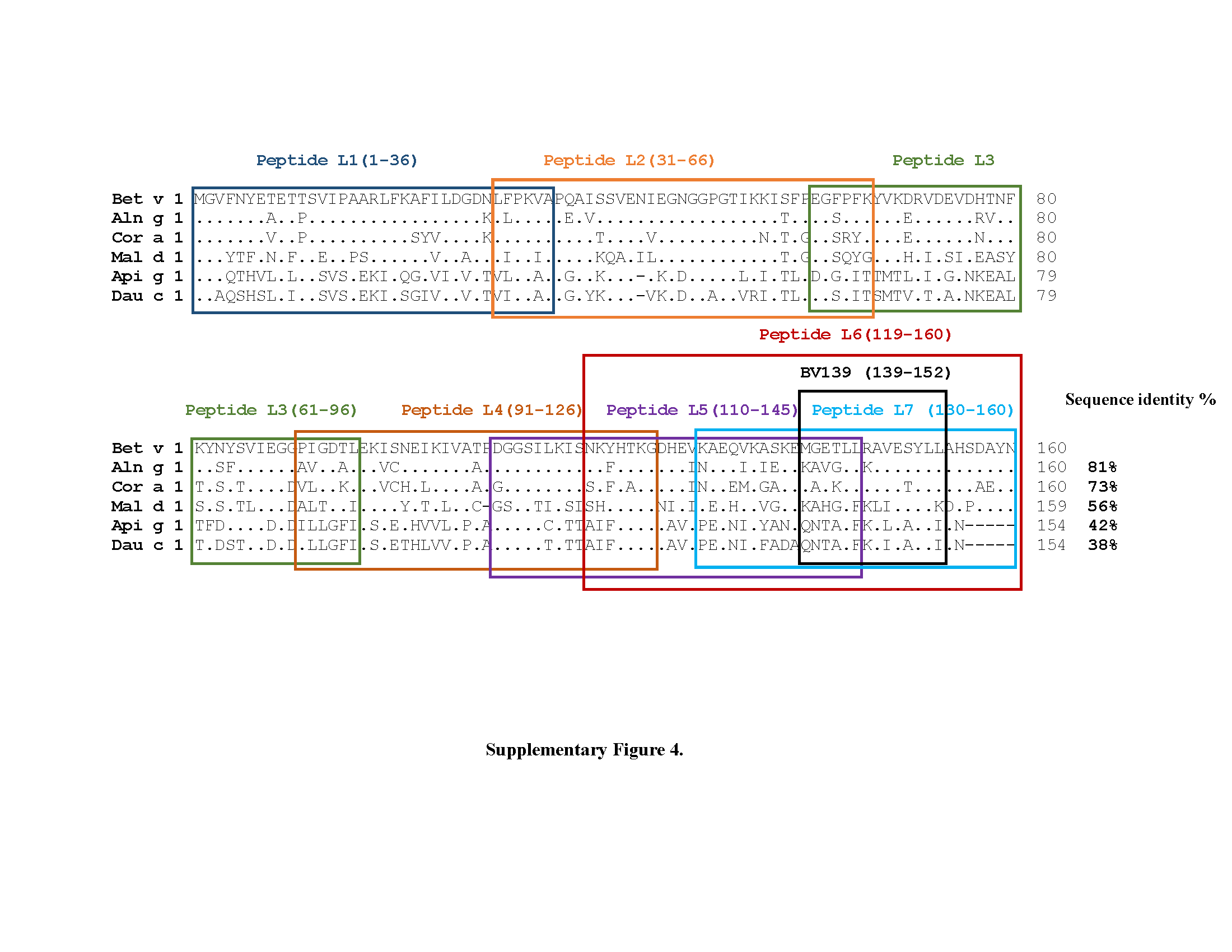

Supplement: Supplementary file 5 [file Image_4.tiff]

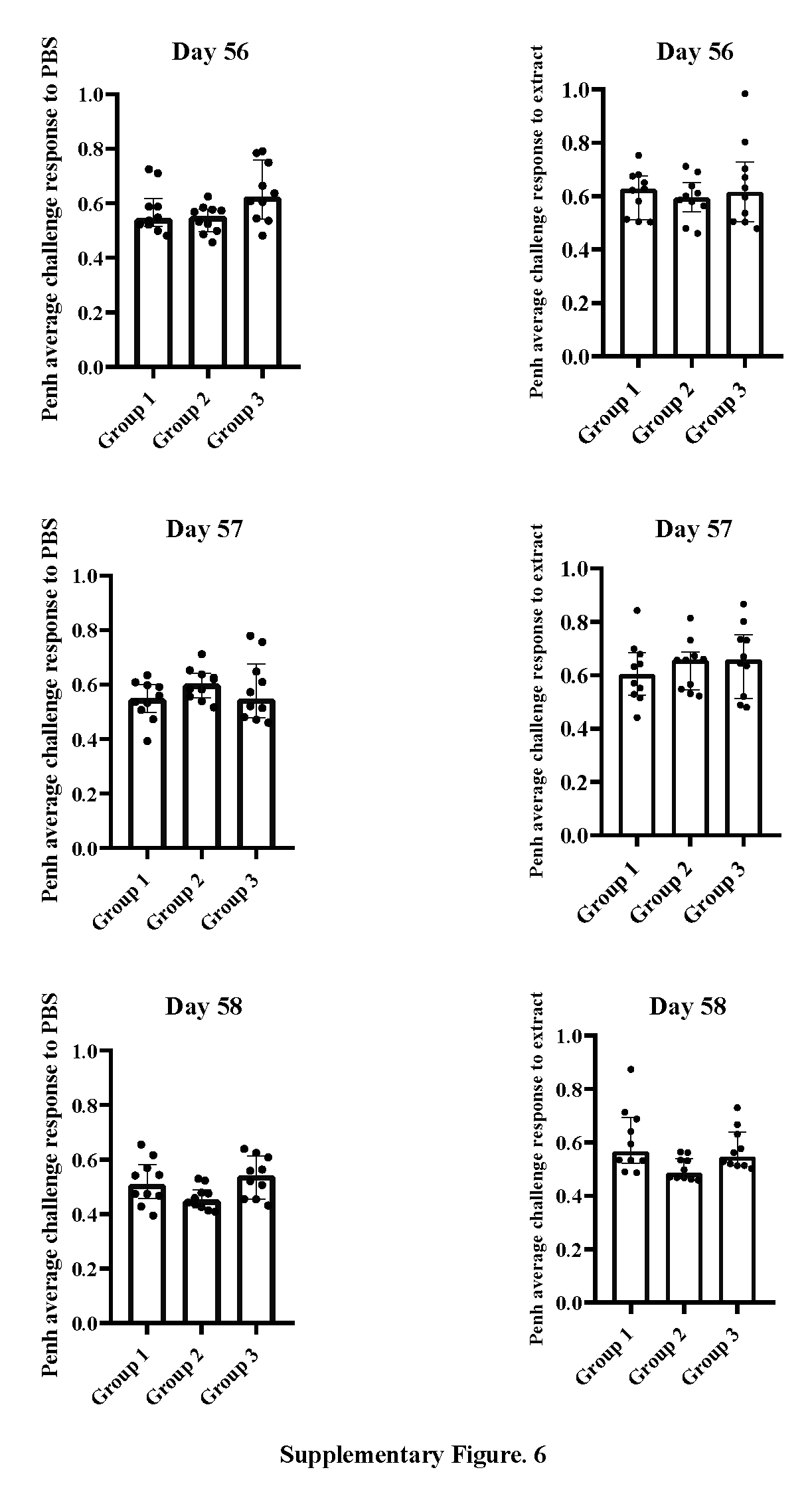

Supplement: Supplementary file 7 [file Image_6.tiff]
